# Adaptation and Validation of the Parental Stressor Scale: NICU for Spanish Populations

**DOI:** 10.3390/nursrep16070216

**Published:** 2026-06-26

**Authors:** Regina Matey Sánchez, Mónica Riaza Gómez, Miguel A. Reina

**Affiliations:** 1 Escuela de Enfermería, Facultad de Medicina, Universidad CEU San Pablo, 28003 Madrid, Spain; miguelangel@perticone.e.telefonica.net; 2 Unidad de Cuidados Intensivos (UCI), Neonatos y Pediátrica, Hospital Universitario HM Montepríncipe, 28660 Madrid, Spain; 3 Unidad Mixta de Cuidados Intensivos Neonatales y Pediátricos, Clínica Universidad de Navarra, 31008 Pamplona, Spain; mriazagomez@unav.es; 4 Departamento de Medicina, Universidad de Navarra, 31009 Pamplona, Spain; 5 Departamento de Ciencias Médicas Clínicas, Facultad de Medicina, Universidad CEU San Pablo, 28003 Madrid, Spain

**Keywords:** parental stress, psychometric validation, PSS:NICU, cultural adaptation, neonatal intensive care, factor analysis, Spain

## Abstract

**Background/Objectives:** Parental stress during neonatal intensive care unit (NICU) admission affects parental well-being, bonding, and neonatal outcomes. Reliable assessment requires instruments adapted to the linguistic and cultural context of each population. This study aimed to adapt the Parental Stressor Scale: NICU (PSS:NICU) for use in Spain and evaluate its psychometric properties. **Methods:** The adaptation comprised forward translation, back-translation, expert review, pilot testing with cognitive debriefing (*n* = 15), and psychometric evaluation. The adapted scale was administered to 160 parents (80 mothers, 80 fathers) of NICU-admitted neonates; 159 cases were retained for analysis. Internal consistency was assessed with Cronbach’s alpha. Exploratory factor analysis (EFA; maximum likelihood, Promax rotation) with parallel analysis examined the factor structure. Confirmatory factor analysis (CFA) tested the original four-factor model, and a second-order model with five first-order factors was subsequently evaluated. **Results:** Internal consistency was excellent (total α = 0.968; subscales α = 0.866–0.961). Parallel analysis supported a five-factor EFA solution over a four-factor solution. CFA of the original four-factor model yielded poor fit (CFI = 0.755, TLI = 0.737, RMSEA = 0.125). A second-order model with five first-order factors and one general stress factor improved fit (CFI = 0.819, RMSEA = 0.107), though indices remained below conventional thresholds. Standardised factor loadings were moderate to high (0.57–0.96). **Conclusions:** The Spanish PSS:NICU demonstrates excellent reliability and provides preliminary evidence supporting its use for research and clinical assessment. The original four-factor structure was not replicated; the data suggest a five-factor organisation with evidence of a higher-order stress construct. Structural validity requires confirmation in larger, independent samples.

## 1. Introduction

### 1.1. Context

When a newborn is admitted to a neonatal intensive care unit (NICU), the experience places parents under considerable emotional strain. The disruption of expected caregiving routines, the sight of a fragile infant connected to monitoring equipment, and the uncertainty about the child’s prognosis converge to produce a stress response that affects parental well-being, the parent–infant relationship, and, indirectly, neonatal outcomes [1,2]. Parents in NICUs frequently report helplessness, guilt, and anxiety, particularly when physical contact with the child is restricted [3].

Recognising parental stress as a modifiable factor, researchers have demonstrated that structured family-support programmes can reduce anxiety among NICU parents [4] and that active parental involvement—kangaroo care, participation in feeding and daily routines—acts as a buffer [5]. The design and evaluation of such interventions, however, depend on having assessment instruments that are psychometrically sound and culturally adapted to the population of interest [1].

### 1.2. Justification

The Parental Stressor Scale: NICU (PSS:NICU), developed by Miles, Funk, and Carlson [6], is the most widely used measure of parental stress in neonatal intensive care. It has been validated in several countries, including Chile [7] and Mexico [8], with generally consistent psychometric performance across Spanish-speaking populations. Nevertheless, cross-cultural adaptation research has shown that applying an instrument validated in one cultural setting directly to another can compromise measurement accuracy [9]. Differences between Latin American and European Spanish—both lexical and pragmatic—affect item comprehension, and healthcare system characteristics (NICU access policies, staffing models, communication norms) shape how parents perceive and report stress [10].

No version of the PSS:NICU has been validated for the population of Spain. This gap prevents researchers and clinicians from accurately assessing parental stress in Spanish NICUs and from evaluating the effectiveness of locally implemented support strategies [11].

### 1.3. Objectives

This study aimed to produce a Spanish version of the PSS:NICU adapted for the cultural and linguistic context of Spain and to evaluate its psychometric properties. The specific objectives were: (1) to carry out the cultural and linguistic adaptation through a structured process of translation, expert review, and pilot testing; (2) to assess internal consistency; (3) to examine the factor structure through exploratory and confirmatory factor analysis; and (4) to compare the psychometric performance of the Spanish version with that of the Chilean validated version [7].

## 2. Materials and Methods

### 2.1. Study Design

This was a cross-sectional methodological study focused on the cultural adaptation and psychometric evaluation of the PSS:NICU for use in Spain.

### 2.2. Participants

The sample comprised 160 parents (80 mothers, 80 fathers) of neonates admitted to the NICU at Hospital Universitario HM Montepríncipe (Madrid, Spain). Inclusion criteria were: being a parent of a neonate currently hospitalised in the NICU, willingness to participate, and ability to read and respond in Spanish. Parents of neonates with a terminal prognosis and those unable to provide informed consent were excluded. One case was excluded from psychometric analyses due to missing data, yielding an analytic sample of *n* = 159.

### 2.3. Cultural Adaptation Process

The adaptation aimed to achieve linguistic, semantic, and cultural equivalence with the original instrument and followed five phases.

Phase 1—Forward translation. Two independent bilingual translators with expertise in health sciences translated the English-language PSS:NICU into Spanish. The Chilean validated version [7] was consulted as a reference. Discrepancies were resolved by consensus.

Phase 2—Back-translation. A third bilingual translator, with no prior exposure to the instrument, back-translated the reconciled Spanish version into English. The result was compared with the original to identify conceptual divergences.

Phase 3—Expert panel review. A panel of specialists in neonatal care, psychometrics, and linguistics evaluated each item for clarity, cultural relevance, and appropriateness. A notable adaptation identified during this phase was the replacement of “0” (representing “no stress”) with “1” on the Likert scale: Spanish respondents in informal pre-testing consistently read “0” as “not applicable” rather than as a valid low-stress anchor.

Phase 4—Pilot testing. The revised scale was administered to a pilot sample of 15 parents. Cognitive debriefing was conducted to detect items that were ambiguous or difficult to interpret; feedback was incorporated into the final version.

Phase 5—Final version. The definitive Spanish PSS:NICU was established and used for the psychometric evaluation with the full sample.

### 2.4. Assessment Instrument

The adapted PSS:NICU contains 36 items distributed across four subscales as defined in the original instrument [6]: (1) Sights and Sounds (7 items), addressing environmental stressors; (2) Infant Appearance and Behaviour (16 items), covering stressors related to the infant’s physical condition and behavioural responses; (3) Parental Role and Staff Communication (9 items), measuring stress arising from the caregiving role and interactions with the healthcare team; and (4) Parental Role Alteration (4 items), assessing the perceived impact of the NICU experience on the parental role. Responses are recorded on a Likert scale from 1 (low stress) to 5 (high stress). A single global item (P8) assesses overall stress perception independently of the subscales.

### 2.5. Data Collection

Participants completed the adapted scale in a private area within the NICU over a six-month recruitment period. Sociodemographic information (age, gender, educational level) was recorded alongside the scale responses.

### 2.6. Statistical Analysis

The analytical strategy comprised four stages: internal consistency, exploratory factor analysis, confirmatory factor analysis, and evaluation of alternative models.

Internal consistency. Cronbach’s alpha was computed for the total scale and for each of the four originally defined subscales. Values above 0.70 were considered acceptable, above 0.80 good, and above 0.90 excellent [12]. Corrected item–total correlations were computed within each subscale to evaluate item discriminative capacity. Alpha-if-item-deleted analysis was performed to assess the contribution of each item.

Exploratory factor analysis (EFA). EFA was conducted using maximum likelihood extraction with Promax (oblique) rotation, appropriate when factors are expected to correlate [13]. The number of factors to retain was determined by parallel analysis [14], supplemented by eigenvalue inspection and interpretability. Model fit was compared across solutions using RMSEA, TLI, and BIC.

Confirmatory factor analysis (CFA). CFA was used to test the fit of the original four-factor model. Fit was evaluated using RMSEA (≤0.06 good, ≤0.08 acceptable), CFI and TLI (≥0.95 good, ≥0.90 acceptable), and SRMR (≤0.08 good) [15].

Second-order modelling. When the original model showed inadequate fit, a second-order model was specified in which a general stress factor loaded on five first-order factors derived from the empirical structure. This hierarchical approach is consistent with the theoretical conceptualisation of the PSS:NICU as measuring distinct but correlated facets of an overarching parental-stress construct [16].

Descriptive and comparative analyses. Means, standard deviations, and score distributions were calculated for each subscale and for the total scale. Gender differences were examined using independent-samples t-tests with Welch’s correction; effect sizes were computed as Cohen’s *d* (0.20 small, 0.50 medium, 0.80 large).

Software. Descriptive statistics and internal consistency analyses were performed in IBM SPSS Statistics (version 29). Exploratory and confirmatory factor analyses, including second-order modelling, were conducted in RStudio using the *psych* [17] and *lavaan* [18] packages. R version 4.5.3 (R Foundation for Statistical Computing, Vienna, Austria) within RStudio version 2026.01.2+418 (Posit Software, PBC, Boston, MA, USA), using the lavaan package version 0.6-21 and the psych package version 2.6.5.

### 2.7. Ethical Considerations

The study was approved by the Comité Ético de Investigación con Medicamentos (CEIm) of HM Hospitales (protocol 21.04.1837-GHM, 21 July 2021) and conducted in accordance with the Declaration of Helsinki. All participants provided written informed consent.

## 3. Results

### 3.1. Internal Consistency

Cronbach’s alpha for the total 36-item scale was 0.968 (*n* = 159), indicating excellent internal consistency. Subscale values were: Sights and Sounds, α = 0.866 (7 items); Infant Appearance and Behaviour, α = 0.961 (16 items); Parental Role and Staff Communication, α = 0.943 (9 items); Parental Role Alteration, α = 0.920 (4 items). All subscale values exceeded the 0.80 threshold for good reliability. Alpha-if-item-deleted analysis showed that removal of any single item would not increase the alpha of its subscale, indicating that every item contributes to measurement precision. Corrected item–total correlations ranged from 0.53 to 0.88 across all subscales (Table 1), confirming adequate discriminative capacity.

Alpha values were consistent across genders (mothers: α = 0.97; fathers: α = 0.97), with per-subscale differences not exceeding 0.03.

### 3.2. Exploratory Factor Analysis

The Kaiser–Meyer–Olkin measure of sampling adequacy was 0.92 and Bartlett’s test of sphericity was significant (χ^2^ = 6024.624, *df* = 630, *p* < 0.001), indicating that the correlation matrix was suitable for factor analysis.

Parallel analysis suggested retaining five factors. Table 2 compares the four- and five-factor solutions obtained by maximum likelihood extraction with Promax rotation. The five-factor solution showed better fit on all indicators (RMSEA = 0.099 vs. 0.107; TLI = 0.812 vs. 0.784; BIC = −1148.67 vs. −1110.96). The fifth factor emerged primarily from the differentiation of the equipment- and monitoring-related items (P2 group) from the remaining items in the original Sights and Sounds subscale.

The five-factor structure suggests that the NICU stressor space, as captured by the Spanish PSS:NICU, is organised into more differentiated domains than the original four-subscale model assumes. Specifically, the data point to a distinction between items addressing the physical environment and equipment (monitors, alarms, other patients) and items related to visual stressors associated with the infant’s bodily appearance in the NICU setting.

### 3.3. Confirmatory Factor Analysis

A CFA testing the original four-factor model—Sights and Sounds, Infant Appearance and Behaviour, Parental Role and Staff Communication, and Parental Role Alteration—yielded the following fit indices: χ^2^ = 2051.909 (*df* = 588), CFI = 0.755, TLI = 0.737, RMSEA = 0.125, SRMR = 0.098 (Table 3). All indices fell outside the range conventionally accepted for adequate model fit [15], indicating that the a priori four-factor structure did not provide a satisfactory account of the observed covariance pattern in the Spanish sample.

Examination of modification indices revealed substantial local dependencies between conceptually similar item pairs within the same subscale—notably P4.1–P4.2 (speed and vocabulary of clinical explanations), P4.4–P4.5 (definitiveness and sufficiency of information), P6.3–P6.4 (multiple staff members and lack of identification), P2.1–P2.2 (monitors and equipment sounds), P2.2–P2.4 (equipment sounds and presence of other patients), and P5.3–P5.5 (crying/moaning and expressions of pain). Although these dependencies suggest content overlap or shared method variance among semantically related items, no post hoc model respecifications were introduced to preserve the theoretical integrity of the instrument and to avoid capitalising on chance.

### 3.4. Second-Order Factor Model

Given the inadequacy of the original four-factor CFA, a second-order model was evaluated. This model specified five first-order factors—Environment, Infant Appearance, Behavioural/Clinical Stress, Parental Role and Communication, and Parental Role Alteration—loading on a single second-order factor representing general parental stress. The five-factor first-order structure reflected the differentiation observed in the EFA, in which items clustered into finer-grained domains than those defined by the original subscales.

The second-order model fit the data better than the original four-factor CFA: χ^2^ = 1666.609 (*df* = 589), CFI = 0.819, TLI = 0.807, RMSEA = 0.107 (90% CI: 0.101–0.113), SRMR = 0.074 (Table 3). Although this represents a meaningful improvement—particularly in CFI (+0.064), TLI (+0.070), and SRMR (−0.024)—the fit indices remain below the thresholds conventionally recommended for structural confirmation [15].

Standardised factor loadings in the second-order model were predominantly moderate to high across all five first-order factors: Environment (λ ≈ 0.61–0.77), Infant Appearance (λ ≈ 0.57–0.88), Parental Role and Communication (λ ≈ 0.61–0.92), Parental Role Alteration (λ ≈ 0.82–0.93), and Behavioural/Clinical Stress (λ ≈ 0.79–0.96). These loadings indicate that items are meaningfully related to their respective latent factors, even though the global fit of the model does not reach conventional benchmarks.

### 3.5. Descriptive Statistics and Gender Differences

Table 4 presents the descriptive statistics for the subscale and total scores as originally defined.

Parental Role Alteration produced the highest per-item stress (*M* = 2.74), followed by Sights and Sounds (*M* = 2.67) and Infant Appearance and Behaviour (*M* = 2.50). Parental Role and Staff Communication yielded the lowest per-item stress (*M* = 1.94), consistent with a pattern in which stressors directly related to the child’s condition and the disruption of caregiving evoke more distress than communication-related factors.

Fathers reported higher total stress than mothers (*M* = 93.2, SD = 31.6 vs. *M* = 80.9, SD = 31.7; *t* = −2.44, *p* < 0.05, d = 0.39), with significant differences also on Infant Appearance and Behaviour (*t* = −2.58, *p* < 0.05) and Parental Role and Staff Communication (*t* = −2.49, *p* < 0.05). The effect size was small to medium.

### 3.6. Cross-Cultural Comparison

Table 5 summarises the comparison between the Spanish and Chilean [7] versions of the PSS:NICU.

Both versions showed high total-scale reliability (α = 0.968 and 0.932). The most notable difference concerns the factor structure: the Chilean EFA yielded three dimensions, the Spanish EFA five. Both studies identified empirical structures that differed from the original theoretical organisation. Direct comparison of model fit is limited because the two studies used different analytical approaches (CFA in Spain, EFA only in Chile) and reported different fit statistics.

## 4. Discussion

### 4.1. Internal Consistency

The Spanish PSS:NICU demonstrated excellent internal consistency at the total-scale level (α = 0.968) and good to excellent reliability for all four originally defined subscales (range: 0.866–0.961). These values compare favourably with those reported for the Chilean version (α = 0.932 total) [7] and with the broader literature on PSS:NICU adaptations. Reliability was consistent across genders (Δα ≤ 0.03), providing preliminary evidence that the instrument performs similarly for mothers and fathers, although formal measurement invariance testing was not conducted. Corrected item–total correlations (0.53–0.88) and the stability of alpha-if-item-deleted values confirm that every item contributes to measurement precision within its designated subscale.

### 4.2. Factor Structure

The factor-analytic results paint a nuanced picture. On the one hand, the EFA confirmed that parental stress in the NICU is multidimensional, with high factorial adequacy (KMO = 0.92) and a clearly interpretable multi-factor solution. On the other hand, the empirical structure did not map neatly onto the original four subscales.

Parallel analysis and comparative fit indices favoured a five-factor over a four-factor EFA solution. The fifth factor arose from the separation of equipment- and monitoring-related items from the remaining items in the Sights and Sounds subscale, suggesting that these two content domains may function as distinguishable sources of stress. This distinction has clinical plausibility: a parent may habituate to the general sights and sounds of a NICU while remaining acutely stressed by the equipment attached to their own child.

CFA of the a priori four-factor structure yielded poor fit (CFI = 0.755, RMSEA = 0.125), indicating that the original model does not adequately account for the covariance pattern in this sample. Modification indices pointed to local dependencies among semantically related item pairs, but no post hoc respecifications were introduced to avoid overfitting. A second-order model with five first-order factors and a general stress factor improved fit (CFI = 0.819, RMSEA = 0.107), lending some support to the idea of a hierarchical organisation in which specific stressor domains contribute to an overarching parental-stress construct. Nevertheless, the fit indices of this model remain below the thresholds recommended by Hu and Bentler [15] for structural confirmation, and the results should be interpreted as suggestive rather than definitive. Although parallel analysis suggested a five-factor solution, the moderate fit of both the exploratory and confirmatory structures indicates that the dimensionality of the Spanish version remains provisional and requires independent replication.

It is worth noting that the Chilean validation [7] also did not recover the original four-factor model, reporting instead a three-dimensional solution. Taken together, these findings suggest that the internal organisation of the PSS:NICU items may be more fluid across cultural and linguistic contexts than the original subscale structure implies. Rather than indicating a flaw in the instrument, this variability may reflect differences in sample characteristics, clinical contexts, or cultural interpretation of NICU-related stressors.

### 4.3. Comparison with Existing Literature

The stress profile observed in this sample is consistent with previous work. In the Chilean study [7], disruption of the parental role was the principal stressor; the same pattern appeared here (Parental Role Alteration: item *M* = 2.74). A meta-analysis across multiple countries [19] identified sensory overload and role disruption as the dominant stressor categories—a finding that our data corroborate.

Fathers in this sample reported higher total stress than mothers (*d* = 0.39), a finding that runs counter to Latin American multicentre data where mothers scored higher [8]. Two non-exclusive explanations deserve consideration: cultural differences in paternal role expectations within the Spanish healthcare system, and the balanced composition of our sample (50% fathers), which afforded statistical power that studies with predominantly maternal samples often lack. Adama et al. [20] have called for tailored family-support programmes in NICUs; the availability of a locally evaluated stress measure is an essential step toward designing and appraising such programmes in Spain.

### 4.4. Strengths

To our knowledge, this is the first study to adapt and psychometrically evaluate the PSS:NICU in Spain. The balanced sample (80 mothers, 80 fathers) stands out in a field where validation studies typically over-represent mothers, and it permitted a meaningful examination of gender differences. The five-phase adaptation process followed recommended cross-cultural guidelines, and the analytical strategy—combining EFA, CFA, and second-order modelling with modern software (R/lavaan)—goes beyond the approaches used in previous PSS:NICU validations.

### 4.5. Limitations

Several limitations should be weighed when interpreting these results. The sample was drawn by convenience from a single private-sector NICU in Madrid, which may limit generalisability to public hospitals, other regions, or NICUs with different care models. The sample size (*n* = 159; ratio of 4.4 subjects per item) meets the minimum recommended by Sapnas and Zeller [21] and is comparable to the Chilean validation (*N* = 221, 5.8:1), but it falls below the 10:1 ratio suggested by Nunnally [22]. Wolf et al. [23] showed that *N* = 150 provides adequate power for CFA models with three or more indicators per factor and moderate-to-high loadings; however, the suboptimal CFA fit in the present study may partly reflect sample-size limitations. The content validity process, while conducted with experienced NICU professionals, did not include formal quantitative indices (I-CVI, S-CVI). Test–retest reliability was not assessed, precluding conclusions about temporal stability. EFA and CFA were performed on the same sample; although this is an accepted practice in cross-cultural adaptation when the a priori structure is well established [16], independent replication is needed to confirm the observed factor structure. Finally, the gender comparison should be regarded as exploratory, since formal measurement invariance testing requires larger subgroup sizes (*n* ≥ 200 per group) [24].

### 4.6. Recommendations for Future Research

The most immediate next step is a multicentre replication with a larger sample drawn from public and private NICUs across different Spanish regions, which would consolidate the structural evidence and enable measurement invariance testing. Concurrent administration of a validated anxiety or depression measure (e.g., STAI, PHQ-9) would provide the external criterion needed to assess convergent validity. Test–retest data at a two-to-four-week interval are necessary to evaluate temporal stability. Content validity should be revisited with I-CVI and S-CVI methodology applied by an independent expert panel. Finally, the instrument should be used in prospective intervention studies, which would simultaneously generate evidence of responsiveness—a measurement property not assessed here.

### 4.7. Summary of Measurement Properties

Table 6 summarises the evidence for each measurement property assessed, following the COSMIN taxonomy [25].

## 5. Conclusions

This study presents the first cultural adaptation and psychometric evaluation of the Parental Stressor Scale: NICU for the population of Spain. The adapted instrument demonstrates excellent internal consistency (α = 0.968 total; subscale range 0.866–0.961), supporting its use as a reliable measure of parental stress in Spanish NICUs.

The factor-analytic findings indicate that the internal organisation of the PSS:NICU items in the Spanish sample is more differentiated than the original four-factor structure. EFA supported a five-factor solution, and CFA did not confirm the a priori four-factor model. A second-order model with five first-order factors and a general stress factor provided improved, though still incomplete, fit. These results suggest that the construct of parental stress in the NICU, as captured by the PSS:NICU, may be organised hierarchically, with a general stress dimension underpinning more specific stressor domains that do not align exactly with the original subscale boundaries.

The Spanish PSS:NICU provides preliminary evidence supporting its use for clinical screening and research purposes, with scores interpreted at the total-scale level or with reference to the original subscale framework while acknowledging its structural limitations. Additional studies with larger, independent samples are needed to consolidate the structural evidence before definitive recommendations can be made. Further work is needed to assess temporal stability, convergent validity with external criteria, and responsiveness to intervention.

## Figures and Tables

**Table 1:** Item-Level Descriptive Statistics and Corrected Item–Total Correlations (*n* = 159).

Item	Content (Spanish, as Administered)	English Translation (Reference)	*M*	*SD*	*r_it_*
Sights and Sounds (α = 0.866)					
P1.1	Observar el cuerpo hinchado de mi hijo	*Seeing my baby’s swollen body*	2.87	1.41	0.60
P1.2	Cambios de color en la piel	*Changes in my baby’s skin colour*	2.99	1.27	0.70
P1.3	Parecía que mi hijo tenía frío	*My baby seemed to be cold*	2.41	1.14	0.71
P2.1	Ver el funcionamiento de su cuerpo u órganos en los monitores	*Seeing my baby’s bodily or organ functions on the monitors*	2.52	1.25	0.61
P2.2	El sonido de los monitores y equipos	*The sound of the monitors and equipment*	2.76	1.11	0.72
P2.3	Los sonidos repentinos de las alarmas	*The sudden sounds of the alarms*	2.19	1.13	0.54
P2.4	La presencia de otros niños enfermos en la unidad	*The presence of other sick infants in the unit*	3.00	1.20	0.62
Infant Appearance and Behaviour (α = 0.961)					
P3.1	Presencia de tubos y sondas en mi hijo	*The presence of tubes and catheters on my baby*	3.13	1.40	0.66
P3.2	Aspiración de secreciones u otros líquidos	*Suctioning of secretions or other fluids*	2.61	1.31	0.75
P3.3	Uso de agujas en mi hijo	*The use of needles on my baby*	2.82	1.40	0.73
P3.4	Hacer que mi hijo tosa, respire fuerte y profundamente	*Making my baby cough and breathe hard and deeply*	2.57	1.39	0.75
P3.5	Que una máquina respire por mi hijo	*Having a machine breathe for my baby*	2.99	1.64	0.70
P3.6	Cortes o herida operatoria en mi hijo	*Cuts or a surgical wound on my baby*	2.57	1.42	0.79
P3.7	Inyecciones/vacunas	*Injections/vaccinations*	2.08	1.19	0.53
P5.1	Confuso o desorientado	*(My baby being) confused or disoriented*	2.28	1.30	0.83
P5.2	Comportamiento rebelde o de poca cooperación	*Uncooperative or resistant behaviour*	2.06	1.15	0.76
P5.3	Llanto y quejidos	*Crying and moaning*	2.55	1.25	0.77
P5.4	Demandante (muy requiriente de atención)	*Demanding (requiring a great deal of attention)*	2.60	1.37	0.85
P5.5	Mostrando o evidenciando dolor	*Showing or displaying pain*	2.54	1.26	0.82
P5.6	Inquietud o intranquilidad	*Restlessness or agitation*	2.42	1.40	0.79
P5.7	Incapacidad para hablar o llorar	*Inability to speak or cry*	2.29	1.33	0.87
P5.8	Miedo	*Fear*	2.23	1.26	0.86
P5.9	Rabia	*Anger*	2.29	1.36	0.84
Parental Role and Staff Communication (α = 0.943)					
P4.1	Explicaban muy rápido	*They explained things too quickly*	1.91	1.10	0.87
P4.2	Usaban palabras que no comprendía	*They used words I did not understand*	1.84	1.04	0.79
P4.3	Me decían versiones distintas sobre la condición de mi hijo	*They gave me different versions of my baby’s condition*	2.14	1.29	0.76
P4.4	No entregaban una versión definitiva de lo que le ocurría a mi hijo	*They did not give a definitive account of what was happening to my baby*	2.14	1.34	0.86
P4.5	No hablaban lo suficiente conmigo	*They did not talk with me enough*	2.06	1.35	0.86
P6.1	Estar bromeando, riendo o hablando fuerte	*Staff joking, laughing, or talking loudly*	1.95	1.20	0.63
P6.2	No conversaban lo suficiente conmigo	*They did not converse with me enough*	1.84	1.14	0.82
P6.3	Distintas personas hablando conmigo	*Different people talking to me*	1.71	0.98	0.71
P6.4	No indicarme sus nombres o quienes eran al atender a mi hijo	*Not telling me their names or who they were when caring for my baby*	1.82	1.18	0.75
Parental Role Alteration (α = 0.920)					
P7.1	No poder cuidar a mi hijo yo mismo	*Not being able to care for my baby myself*	3.01	1.49	0.79
P7.2	No poder visitar a mi hijo cuando yo quiero	*Not being able to visit my baby whenever I want*	2.47	1.36	0.77
P7.3	No poder estar cuando mi hijo está llorando	*Not being able to be there when my baby is crying*	2.67	1.48	0.88
P7.4	No poder sostener o tomar en brazos a mi hijo	*Not being able to hold or cradle my baby*	2.81	1.48	0.83

Note: *M* = mean; *SD* = standard deviation; *r_it_* = corrected item–total correlation within the originally assigned subscale. Scale range: 1 (low stress) to 5 (high stress). Alpha values reported for the four subscales as defined in the original PSS:NICU. The items were administered in Spanish; the Spanish wording is the validated instrument and the object of measurement, and the reported statistics correspond to that wording. The English column is a reference translation of the original PSS:NICU items (Miles, Funk & Carlson) provided for reader comprehension only and was not the form administered to participants.

**Table 2:** Comparison of EFA Solutions (Maximum Likelihood, Promax Rotation).

Solution	RMSEA	TLI	BIC
Four factors	0.107	0.784	−1110.96
Five factors	0.099	0.812	−1148.67

**Table 3:** Model Fit Indices: CFA and Second-Order Model.

Model	χ^2^	df	CFI	TLI	RMSEA	90% CI RMSEA	SRMR
Four-factor CFA (original)	2051.909	588	0.755	0.737	0.125	—	0.098
Second-order (5 first-order + general)	1666.609	589	0.819	0.807	0.107	0.101–0.113	0.074

**Table 4:** Descriptive Statistics for PSS:NICU Subscale and Total Scores (*n* = 159).

Subscale	Items	*M*	SD	Observed Range	Item *M*
Sights and Sounds	7	18.72	6.35	7–35	2.67
Infant Appearance and Behaviour	16	40.00	17.11	16–80	2.50
Parental Role and Staff Communication	9	17.42	8.83	9–45	1.94
Parental Role Alteration	4	10.94	5.22	4–20	2.74
Total Scale	36	87.09	32.15	36–180	2.42
Overall Stress (P8)	1	2.91	1.20	1–5	2.91

Note: Item *M* = subscale mean/number of items. P8 is a single global item scored independently.

**Table 5:** Psychometric Comparison: Spanish and Chilean Versions of the PSS:NICU.

Metric	Spanish Version	Chilean Version [7]
Sample size	159	221
Number of items	36 (+1 global)	38 (+1 open-ended)
Cronbach’s α (total)	0.968	0.932
EFA factor solution	5 factors	3 dimensions
Variance explained (EFA)	—	48.9%
KMO	0.92	>0.80 ^a^
CFA/fit index	RMSEA = 0.125 (4-factor)	RMSR = 0.06
Subscale α range	0.866–0.961	0.703–0.904
Likert scale	1–5	0–5
Analytical software	SPSS, RStudio (lavaan, psych)	FACTOR

Note: ^a^ Approximate value; the Chilean study reported KMO > 0.80 [7]. RMSR = Root Mean Square of Residuals. The Chilean study used only EFA (no CFA). The three Chilean dimensions were Clinical (α = 0.885/0.904), Emotional (α = 0.892/0.902), and Communication (α = 0.703).

**Table 6:** Summary of Measurement Properties (COSMIN Framework).

Property	Evidence	Rating	Limitations
Content validity	Expert panel; pilot with cognitive debriefing (*n* = 15); 5-phase adaptation	Moderate	No formal I-CVI/S-CVI
Structural validity	CFA: poor fit for 4-factor model; second-order 5-factor model: improved but below thresholds; EFA: 5 factors supported	Preliminary	Original structure not confirmed; single sample; all fit indices below recommended thresholds
Internal consistency	α = 0.866–0.968; *r*_it_ = 0.53–0.88; alpha-if-deleted stable	Sufficient	—
Cross-cultural validity	Comparable total α with Chilean version; both diverge from original structure	Moderate	Different factor solutions (5 vs. 3 vs. 4 original)
Convergent validity (external)	Not assessed	Indeterminate	Requires concurrent criterion measure
Test–retest reliability	Not assessed	Indeterminate	Requires longitudinal design
Measurement error	Not assessed	Indeterminate	Requires test–retest data
Responsiveness	Not assessed	Indeterminate	Requires intervention study

## Data Availability

The data presented in this study are available on request from the corresponding author. The data are not publicly available due to privacy restrictions.

## Public Involvement Statement

No public involvement in any aspect of this research.

## Guidelines and Standards Statement

This manuscript was drafted following the COSMIN (Consensus-based Standards for the selection of health Measurement Instruments) guidelines for studies on measurement properties.
